# Chronic anthropogenic disturbance mediates the bottom‐up influence of plant diversity on arthropods in tropical forests

**DOI:** 10.1002/ece3.11055

**Published:** 2024-05-13

**Authors:** Orou G. Gaoue, Hermann Bassoki, Anicet G. Dassou

**Affiliations:** ^1^ Department of Ecology and Evolutionary Biology University of Tennessee Knoxville Tennessee USA; ^2^ Faculty of Agronomy University of Parakou Parakou Benin; ^3^ Department of Geography, Environmental Management and Energy Sciences University of Johannesburg Johannesburg South Africa; ^4^ National School of Biosciences and Applied Biotechnology (ENSBBA) National University of Sciences, Technology, Engineering and Mathematics Dassa Benin

**Keywords:** arthropods, biodiversity, bottom‐up processes, herbivory, logging, plant–insect interactions, pruning, tropical trees

## Abstract

Understanding how primary productivity and diversity affect secondary productivity is an important debate in ecology with implications for biodiversity conservation. Particularly, how plant diversity influences arthropod diversity contributes to our understanding of trophic cascades and species coexistence. Previous studies show a positive correlation between plant and arthropod diversity. The theory of associational resistance suggests that plant herbivory rate will decrease with increasing plant diversity indicating feedbacks between primary diversity, productivity, and secondary productivity rates. However, our understanding of how these relations are mediated by anthropogenic disturbance is still limited. We surveyed 10 forest sites, half of which are disturbed by fire, logging, and tree pruning, distributed in two climatic zones in Benin, West Africa. We established 100 transects to record plant species and sampled arthropods using pitfall traps, ceramic plates with bait, and sweeping nets. We developed a structural equation model to test the mediating effect of chronic anthropogenic disturbance on plant diversity and how it influences arthropod diversity and abundance. Arthropod diversity increased but arthropod abundance decreased with increasing intensity of disturbance. We found no significant bottom‐up influence of the plant diversity on arthropod diversity but a significant plant diversity–arthropod abundance relationship. Some arthropod guilds were significantly affected by plant diversity. Finally, herbivory rates were positively associated with arthropod diversity. *Synthesis*. Our results highlight how chronic anthropogenic disturbance can mediate the functional links between trophic levels in terms of diversity and productivity. Our study demonstrated a decoupled response of arthropod diversity and abundance to disturbance. The direct positive influence of plant diversity on herbivory rates we found in our study provides counter‐support for the theory of associational resistance.

## Graphical abstract

How plant diversity influences arthropod diversity contributes to our understanding of trophic cascades and species coexistence. This relationship can be altered by disturbance. We showed that the bottom‐up effect of plant diversity on arthropod diversity and abundance can be decoupled, whereby plant diversity affects arthropod abundance without altering their diversity.

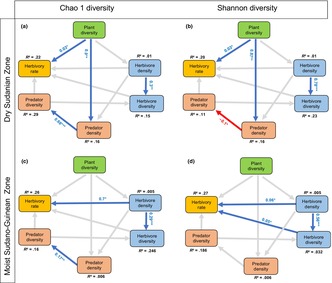

## 1 INTRODUCTION

Tropical forests are known for their high biodiversity which accumulates biomass and serves as an important reservoir of carbon (Staab et al., 2021; Staab & Schuldt, 2020). Maintaining biodiversity in these tropical regions is of paramount importance because it is a major determinant of the dynamics and functioning of communities and ecosystems (Tilman et al., 2014). Understanding the mechanisms by which such diversity is generated and maintained or altered by humans is an ongoing discussion in ecology (Song et al., 2021). Species interactions, particularly between trophic levels, underlie species coexistence and hence the maintenance of biodiversity (Chesson, 2000; Cordonnier et al., 2018; Valladares et al., 2015).

Arthropods, which include insects and spiders and make up about 70% of all forest species, play a key role in the functioning of ecosystems, in food webs, litter decomposition processes, and plant reproduction such as pollination, seed production, and dispersal (Weisser & Siemann, 2008, p. 200). Linking the diversity of plants and arthropods is central to our understanding of species coexistence (Comita & Stump, 2020). Studies on the link between plant and arthropod diversity include manipulative experiments on herbaceous plants (Symstad et al., 2000) and in forest environments (Levi et al., 2019) to show the interactions between arthropods and plants in different plant configurations. Recent studies showed that plant phylogenetic diversity, not species richness, is positively correlated with predator arthropod diversity, and negatively correlated with arthropod herbivore diversity but with no significant effect on their abundance (Staab et al., 2021). Previous studies also showed that plant diversity can have a positive effect on arthropod diversity (Dassou & Tixier, 2016; Siemann et al., 1998).

The interaction between plant diversity and arthropod communities could last over time, thus contributing to the stability of ecosystems. A dependence is often established between a mother plant and its offspring which establishes the same trophic relationships with arthropods. The Janzen–Connell theory predicts that the probability of a seedling surviving is a positive function of the distance between seedling and mother plant (Connell, 1971; Janzen, 1970). This effect is due to the top‐down effects of pathogens and herbivores associated with the mother plants which limit the survival of the nearest seedlings, thus causing conspecific negative density dependence (Comita & Stump, 2020). Such conspecific negative density dependence is a key mechanism for species coexistence and the maintenance of biodiversity (Chesson, 2000; Comita & Stump, 2020). A direct prediction of the Janzen–Connell theory is that diverse plant communities will require diverse arthropod communities to exert appropriate top‐down influence in such a way that no unique plant species has high enough fitness to dominate the community. Plant species provide food resources and habitats for predatory arthropods that enhance their ability to control their communities. It is therefore hypothesized that plant diversity positively affects arthropod diversity and abundance. In contrast, the associational resistance theory suggests that plant herbivory rates will decrease with increasing plant diversity (Guyot et al., 2016), owing to the increased search time by herbivores (Castagneyrol et al., 2014; Guyot et al., 2016; Jactel & Brockerhoff, 2007) or the top‐down control of herbivores by the diverse community of predator arthropods such as spiders (Staab et al., 2021; Staab & Schuldt, 2020). Plant diversity reduces the accessibility to host plants and favors non‐herbivore arthropods. However, plant diversity reduction may also reduce leaf biomass available for herbivores (Salazar et al., 2016) because leaf biomass availability can be a strong predictor of herbivore abundance (Whitfeld et al., 2012). Reconciling Janzen–Connell theory and the theory of associational resistance suggests that an equilibrium plant diversity is necessary for stable coexistence. This equilibrium is possible given that arthropod diversity is not necessarily positively associated with herbivory rates (van Klink et al., 2015).

Most of the evidence for the associational resistance theory is biased toward temperate forest plantation systems. Similarly, there is limited empirical support for the Janzen–Connell theory in tropical African forest ecosystems (Comita et al., 2014; Matthesius, 2006; Terborgh, 2013). Consequently, the nature of the relationships between herbivory rates, and the diversity of arthropods and plants remained poorly understood in tropical African forests. Forest ecosystems in Africa are under increasing pressure from chronic anthropogenic disturbance including fire, overharvesting, deforestation, and fragmentation, which alter ecosystem processes including the diversity and abundance of arthropods (Lewis et al., 2015). For example, a comparative study between secondary forests, pastures, and agricultural land shows a sharp reduction in beetle diversity as a result of chronic anthropogenic disturbance (Barlow et al., 2016). In addition, such reduction in functional group diversity can reduce beta and gamma diversity with significant ecological consequences including species invasion and loss of specific ecological interactions (Young et al., 2016). Fires similar to forest fragmentation can change animal assemblages (Malhi et al., 2014). Recent research suggests that anthropogenic activities negatively influence arthropod composition possibly due to fire, drought, logging, and agriculture (Wagner et al., 2021).

Chronic anthropogenic disturbance can alter plant–ant mutualisms (Bruna et al., 2005; Câmara et al., 2018; Piovia‐Scott, 2011; Sensenig et al., 2017). The nature of the effect depends on the kind of disturbance that the ecosystem experiences. For example, simulated hurricane damage, by causing compensatory plant growth and increased production in rewards, ultimately strengthened plant–ant mutualism in buttonwood mangroves (Piovia‐Scott, 2011). In contrast, fire disrupted ant–plant mutualism in a myrmecophyte‐dominated African savanna by promoting a weaker mutualistic ant species than the strong mutualist *Crematogaster mimosae* (Sensenig et al., 2017). Similarly, livestock grazing can alter ant–plant network structure (Câmara et al., 2018). In contrast, forest fragmentation had no effect on ant diversity and ant–plant mutualistic relationship (Bruna et al., 2005). However, we still have a limited understanding of the role of human activities on plant–arthropod interactions (Comita & Stump, 2020).

In this study, we used a long‐running study system in rarely studied African ecosystems where the disturbance regime has been tracked over several years (Gaoue, 2016; Gaoue et al., 2019; Gaoue & Ticktin, 2007, 2010). These communities are subject to different degrees and nature of disturbance (barking, pruning, logging, and fire) from humans (Gaoue & Ticktin, 2007). We used structural equation modeling (SEM) to investigate how chronic anthropogenic disturbance can alter the bottom‐up influence of plant diversity and abundance on arthropod diversity and abundance. We also investigated the implications for the top‐down effect of arthropod communities in plant biomass via herbivory. We hypothesized that plant diversity has a positive bottom‐up effect on arthropod abundance and diversity. Several mechanisms explain the bottom‐up effects of plant diversity and the top‐down effects of predators. A diverse plant community can provide a diversity of food sources, which can indirectly affect species diversity and secondary productivity in higher trophic levels (Power, 1992). We also hypothesized that diverse and abundant arthropod communities will exert a top‐down effect on plant communities by increasing plant herbivory rates. In ecosystems with high species diversity, predators can feed on the most abundant herbivores, thus improving their fitness and predation success. Connectivity (i.e. consumption intensity) between trophic groups is therefore crucial for controlling herbivores. We hypothesized that anthropogenic disturbance will decrease the diversity and abundance of arthropods and reduce top‐down herbivory effects.

## 2 MATERIALS AND METHODS

### 2.1 Study system

**FIGURE 1:**
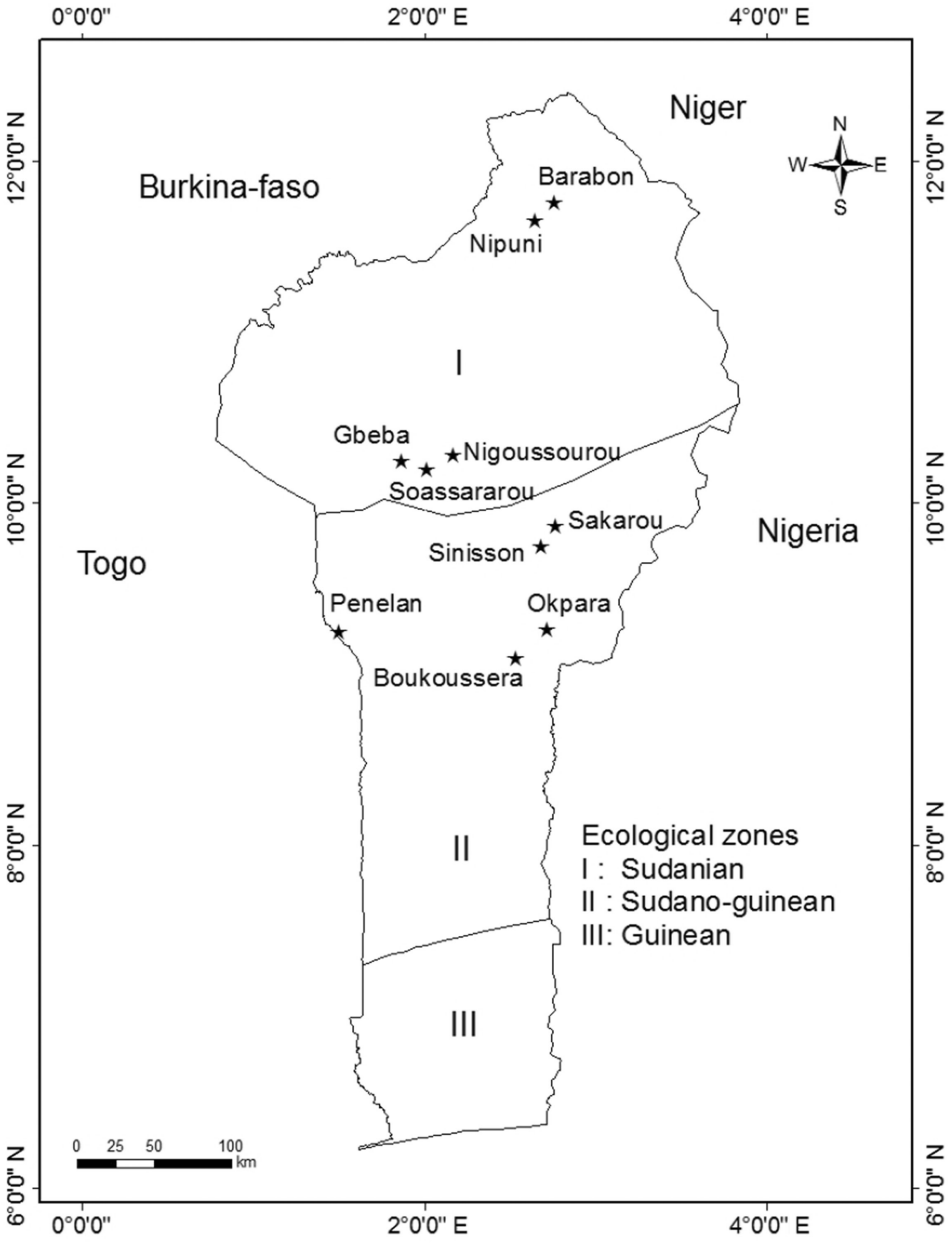
Distribution of study sites across the dry Sudanian and moist Sudano‐Guinean climatic zones in Benin, West Africa. Five populations with various disturbance intensities were sampled in each climatic zone.

Our study was conducted in two climatic zones in northern Benin (Table 1): the dry Sudanian zone (9°45′ N and 12°25′ N) and the moist Sudano‐Guinean zone (7°30′ N and 9°30′ N). The Sudanian zone has a unimodal rainfall regime from May to October with an annual rainfall of up to 1050 mm, a relative humidity ranging from 18% to 99% and the temperature varying from 24 to 31°C (Adomou, 2005). This zone is dominated by hydromorphic soils, laterite cuirasses, and lithosols with a vegetation composed of savannas and especially gallery forests. We sampled five sites in the Sudanian zone: Barabon, Nipuni, Gbeba, Nigoussourou, and Soassararou (Figure 1). The Sudano‐Guinean zone also has a unimodal climate regime with rainfall distributed from May to October with an annual rainfall varying from 900 to 1110 mm (Adomou, 2005). The relative humidity varies from 31% to 98% while the temperature varies between 25 and 29°C. The soils are ferruginous, with a vegetation composed of open forests, dense dry forests, semi‐deciduous dense humid forests, tree, and shrub savannahs. In the Sudano‐Guinean zone, we sampled five sites including Boukoussera, Okpara, Sakarou, Sinisson, and Penelan (Figure 1). All the sites were dominated by *Khaya senegalensis* (Meliaceae) trees. In the Sudanian zone, the sites were all gallery forests while those in the Sudano‐Guinean zone were woodlands and dense dry forests.

**TABLE 1:** Ten independent sites were sampled across two climatic zones (dry Sudanian and moist Sudano‐Guinean zones) and different levels of chronic anthropogenic disturbance.

Climatic zones	Sites	Coordinates	Habitat	Tree pruning	Fire	Logging	Disturbance intensity
Sudanian	Barabon	11°45 N, 2°45 E	Gallery forest	−	−	−	Low
	Nipuni	11°39 N, 2°39 E	Gallery forest	−	+	−	Medium
	Gbeba	10°15 N, 1°52 E	Gallery forest	+	+	+	High
	Nigoussourou	10°17 N, 2°10 E	Gallery forest	+	+	+	High
	Soassararou	10°12 N, 2°01 E	Gallery forest	+	+	+	High
Sudano‐Guinean	Boukoussera	09°06 N, 2°32 E	Dry forest	−	−	+	Medium
	Okpara	09°16 N, 2°43 E	Woodland forest	+	+	+	High
	Sakarou	09°52 N, 2°46 E	Dry forest	+	+	+	High
	Sinisson	09°45 N, 2°41 E	Woodland forest	+	+	+	High
	Penelan	09°15 N, 1°30 E	Gallery forest	−	+	−	Medium

*Note*: Disturbance included tree pruning, fire, logging, and each disturbance type was scored as present (+) or absent (−).

### 2.2 Estimating plant diversity

In each of the 10 sites, we installed 10 transects of 5 m × 50 m every 15 m. These transects were established to survey all plant species and estimate species richness and diversity. In each transect, we identified each plant species including trees, shrubs, and herbs except for grasses. There was no liana in these study sites. For each species, we estimated their cover at the transect level which allowed us to estimate the relative cover percentage for each species. We used these data to estimate the transect‐level species richness and diversity using the Chao 1 abundance‐based estimator (Chao, 1984; Chao & Chiu, 2016). This index estimates the real diversity from an incomplete inventory based on abundance (Gotelli & Colwell, 2011). To estimate herbivory rates, we randomly selected five individual plants per species in each transect. On each plant, we randomly sampled five leaves. We visually inspected each leaf for damage caused by arthropods. In the absence of a leaf area meter, one of us estimated the proportion of the missing leaf biomass area for every species by considering each leaf blade as an ellipse that was divided into four parts along the midrib and the perpendicular line at the middle of the leaf. Biomass loss was estimated for each leaf quadrat to improve precision. Herbivory rate was estimated between 0% and 100% of total leaf area lost and averaged at the plant and transect levels. Leaf herbivory was identified according to the following classification: 0% = no leaf damage; 20% = little damage (punctures and/or small holes) that altogether covers less than one quarter (parts) of the ellipse; 40% = medium damage (a few larger holes that make up nearly two‐quarters of the ellipse); 60% = significant damage (large holes with often larger leaf edge areas eaten away); 80% = very heavy damage (many larger holes and/or larger leaf edge areas eaten with less than a quarter of blade remaining); and 100% = total damage (leaves destroyed and non‐functional).

### 2.3 Estimating arthropod abundance and diversity

To estimate arthropod abundance and diversity, in each transect, we installed, pitfall traps, ceramic plates with bait, and used sweep nets. We combined these three sampling methods to maximize the sampling effort considering the different behaviors and habitats of arthropods (Wynne et al., 2018). We used a 1.5‐m long sweep net with a 30 cm net diameter for 5 min to collect arthropods from various strata and habitats and thus cover up to nearly 90% of the arthropod species in a given site (Viana‐Junior et al., 2021). Each transect was swept by the net for 5 min. “Pitfall” traps or pit traps, a widely used sampling technique, were used to collect mobile arthropods on the ground (Nageleisen & Bouget, 2009). Pitfall traps containing soapy water were installed and left on the site for 24 h, before collecting arthropods that were trapped in the plastic pit. One pitfall trap was installed per transect and placed in the middle of the transect. In each transect, we installed one ceramic plate with bait made of tuna mixed with honey. This type of trap, installed to detect the diversity and abundance of ants, was deployed for 30 minutes before collecting the ants. Baits are stimuli that essentially attract ground‐dwelling ants or any other predators (Nageleisen & Bouget, 2009). Arthropods attracted to the bait were caught using an aspirator for 5 min per trap. All bait traps were set in the morning between 8 and 12 am to prevent ants from being disturbed by the bright sunlight in the afternoon. Ants were counted and collected for later identification at the laboratory. All the arthropods we collected were preserved in 70% alcohol and transported to the laboratory for sorting. The samples were sorted for species identification at the International Institute of Tropical Agriculture insect museum in Abomey‐Calavi, Benin. Arthropod diversity at the transect level was estimated using Chao 1 estimator, similarly to the plant diversity, by using the relative abundance of each arthropod species. Arthropod abundance was estimated at the transect level, first as the number of arthropods for each species, then for each of the three sampling methods, and finally as the total number of individual arthropods for each species considering all sampling methods combined.

### 2.4 Estimating chronic anthropogenic disturbance

To assess the level of disturbance at each site, we considered three criteria are as follows: pruning/debarking, fire, and logging, which are anthropogenic disturbances known to influence arthropod composition (Murphy et al., 2020). We recorded the disturbance level for each site. Pruning and debarking were observed respectively at the level of branches and tree trunks following the method previously developed on these sites (Gaoue & Ticktin, 2007, 2010). We determined whether a site was burned or not by noting the presence of burnt debris. When a site is burned, fire tended to cover the whole site given that it spreads quickly across the dry vegetation. We looked for tree stumps to estimate the intensity of timber logging in each site. The disturbance intensity was estimated as low, medium, and high, based on the extent of tree pruning, logging, and fire presence in each site (Table 1).

### 2.5 Statistical analysis

To estimate plant diversity, we constructed abundance matrices and used the *BiodiversityR* (Kindt, 2020) and *vegan* (Oksanen et al., 2009) packages in R version 4.0.3 (R Core Team, 2021) to calculate the Chao 1 diversity index. Arthropod diversity was estimated as the Shannon diversity index using the *SpadeR* package (Chao et al., 2016). We tested the effects of disturbance and climate on arthropod diversity and abundance using generalized linear models. To test the effect of the diversity and abundance of arthropods on the herbivory rate, we used a generalized linear model with a beta error structure using the package *betareg* (Cribari‐Neto & Zeileis, 2010) given that the response variable, herbivory rate, is a proportion data. Several candidate models were built and the best one was selected based on their Akaike information criterion (AIC). To test the effect of plant diversity on arthropod diversity and abundance, we used a generalized linear model with a Poisson error structure because the response variables are count data. To test the direct and indirect relationships between these variables and highlight guild‐specific responses, we developed a SEM using the *lavaan* package in R (Rosseel, 2012). We first developed an initial model and conducted a goodness‐of‐fit test using the model's chi‐squared and associated *p*‐values to decide whether there were missing paths that needed to be added to our model. We then used the *modificationIndices* function to identify missing paths to include in our model to improve its fit. Our final model was selected when we obtained a non‐significant *p*‐value (*p* > .05) associated with the Minimum Function Test Statistics indicating the best fit between the theoretical and observed variance–covariance matrix (Grace, 2006; Rosseel, 2012).

## 3 RESULTS

We captured 7156 individuals of arthropods belonging to 80 species as compared to the 123.12 species predicted by the Chao1 index, indicating that 43.12 species were not captured. A total of 1064 arthropod individuals (15%) were captured using pitfalls against 5830 (83%) using ceramic plates with bait and 144 (2%) were captured using the sweeping nets. The most abundant arthropod species were *Pheidole* sp. (18%), *Monomorium* sp. (17%), *Monomorium bicolor* (14%), *Oecophylla longinoda* (7%), and *Myrmicaria striata* (7%). Shannon diversity index for arthropods differed significantly between zones (*β* ± SE = 0.353 ± 0.116, *p* = .003), but not between sites (*β* = 0.208 ± 0.112, *p* = .066). We recorded 65 plant species out of the 76.96 species predicted by Chao 1 richness.

### 3.1 Effect of the disturbance on the diversity and abundance of arthropods

**FIGURE 2:**
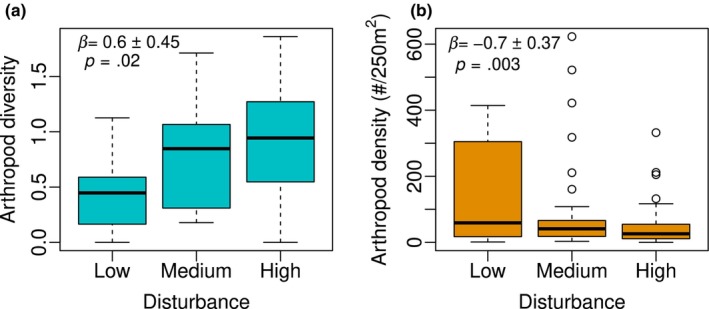
Effect of chronic anthropogenic disturbance on arthropod (a) Shannon diversity and (b) density (plant/250 m^2^) per transect. Arthropod diversity increased with disturbance (a) while disturbance reduced arthropod abundance (b). Disturbance also reduced the variance of arthropod abundance. Arthropod diversity and density were estimated using a combination of three sampling methods including pitfall traps, ceramic plates with baits, and sweeping nets.

Site disturbance was significantly associated with arthropod diversity when estimated as the Shannon diversity index. However, we found no significant association between disturbance and the Chao1 index indicating that disturbance had no influence on species richness but influenced within‐species abundance and overall evenness. Shannon diversity index for arthropods was significantly higher in sites with medium (*β* = 0.552 ± 0.260, *p* = .037, Figure 2a) and high disturbance (*β* = 0.643 ± 0.249, *p* = .011, Figure 2a) than in low disturbance sites, suggesting a positive response of arthropod diversity to disturbance. However, arthropod density was significantly lower in high (*β* = −1.140 ± 0.384, *p* = .004, Figure 2b) than low disturbance sites, but we found no significant difference in arthropod density between low and medium disturbance sites (*β* = −0.284 ± 0.374, *p* = .450, Figure 2b). Overall, these results suggest a decoupled response of arthropod diversity and density to disturbance. While disturbance positively influences arthropod diversity, it reduces their abundance.

### 3.2 Effect of plant diversity on arthropod diversity and abundance

**FIGURE 3:**
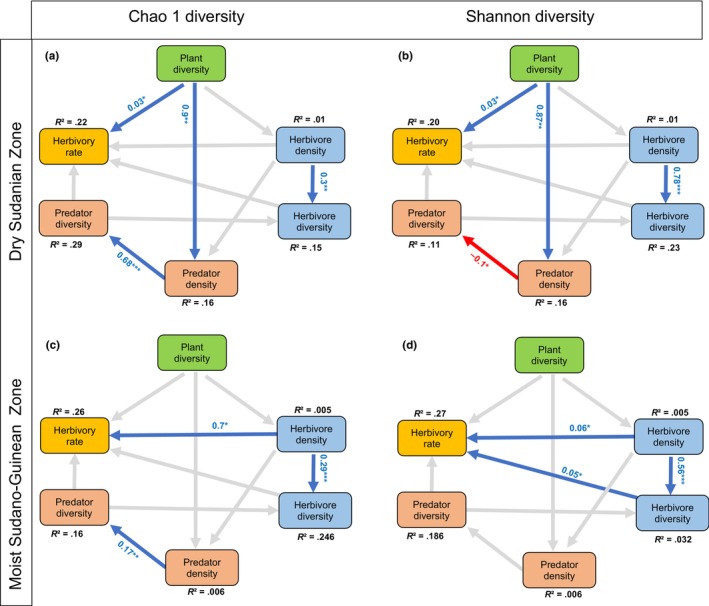
Direct and indirect relationships between plant diversity, herbivory, and arthropod diversity in the Sudanian zone (a, b), with arthropod diversity as (a) the Chao1 diversity and (b) Shannon diversity index, and in the Sudano‐Guinean zone (c, d) with arthropod diversity as (c) the Chao1 index and (d) the Shannon index of diversity. Gray lines represent non‐significant paths in the structural equation model, and blue lines are significant positive relationships and red lines are negative relationships. Asterisks on path coefficients indicate how significance: *p < .05; **p < .001; ***p < .0001.

We found no significant association between plant diversity and arthropod diversity measured as the Chao1 index (*β* = 0.004 ± 0.010, *p* = .713) and Shannon diversity index (*β* = −0.001 ± 0.011, *p* = .930, Figure 3). This lack of a global association between plant diversity and arthropod diversity masked guild‐specific response. Plant diversity was not significantly associated with arthropod herbivore abundance in all climatic zones (Figure 3). However, plant diversity was significantly positively associated with arthropod predator abundance in the dry Sudanian zone (*β* = 0.087 ± 0.028, *p* = .002, Figure 3a,b) but not in the moist Sudano‐Guinean zone (Figure 3c,d). We also found a significant positive direct association between arthropod abundance and diversity for both herbivores and predators (*β* = 0.678 ± 0.171, *p* < .001, Figure 3a–c), except in the Sudano‐Guinean zone when we used the Shannon index as a metric of diversity (Figure 3d). This suggests that plant diversity indirectly positively influenced arthropod predator diversity as mediated by their abundance. Overall, the main difference between the climatic zones was related to the strength of the species interactions. The indirect effects of plant diversity on arthropod abundance and diversity were stronger in the dry Sudanian zone than in the moist Sudano‐Guinean zone (Figure 3).

### 3.3 Effect of arthropod diversity and abundance on herbivory rates

**FIGURE 4:**
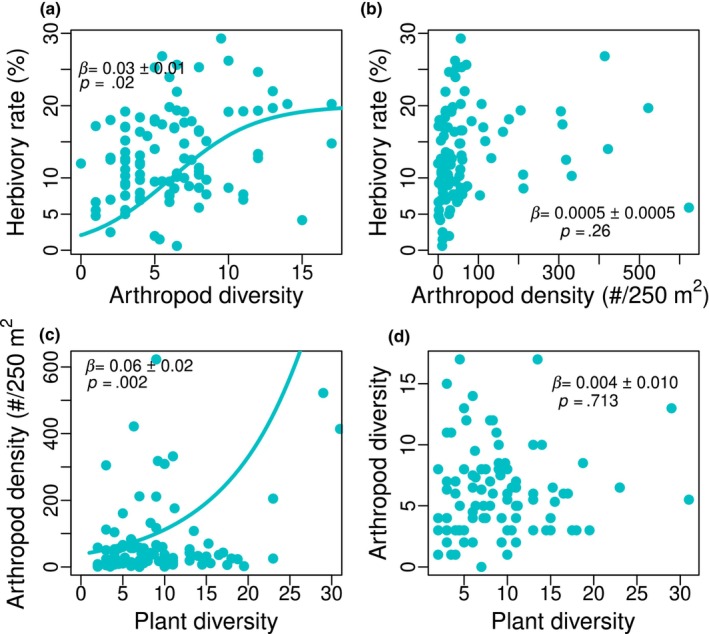
Effects of (a) Shannon diversity index per transect and (b) arthropod density (#/250 m^2^) on herbivory rates; and effects of plant diversity on (c) arthropod density and (d) arthorpod diversity. Arthropod diversit and density were estimated using a combination of three sampling methods including pitfall traps, ceramic plates with baits, and sweeping nets.

Herbivory rate was significantly associated with arthropod diversity, but not with arthropod abundance. Our best model included an additional effect of the Chao 1 arthropod diversity and abundance on herbivory rate (AIC = −271). Herbivory rates increased significantly with arthropod diversity (*β* = 0.035 ± 0.015, *p* = .023, Figure 4a), suggesting trophic niche partitioning within the arthropod communities. Instead, arthropod density also increased with plant herbivory rates, but this association was not significant (*β* = 0.001 ± 0.001, *p* = .265, Figure 4b). Our SEM showed that such influence was due to arthropod herbivore density rather than predators. We found a positive significant association between plant diversity and herbivory rates in the dry Sudanian zone (*β* = 0.026 ± 0.012, *p* = .027, Figure 3a,b) but not in the moist Sudano‐Guinean zone (Figure 3c,d). However, we found a significant positive association between herbivore density and herbivory rate in the moist Sudano‐Guinean zone (*β* = 0.067 ± 0.027, *p* = .015, Figure 3c,d) but not in the dry Sudanian zone (Figure 3a,b).

## 4 DISCUSSION

We investigated the mediating effect of chronic anthropogenic disturbance on plant diversity and its influence on arthropod diversity and abundance. We found no significant association between plant diversity and arthropod diversity but a significant association between plant diversity and the abundance of predator arthropod indicating a guild‐specific bottom‐up response. However, contrary to expectation, we found higher arthropod diversity in highly disturbed sites than in low disturbance sites while arthropod abundance was reduced by disturbance. In addition, arthropod diversity was significantly associated with herbivory rates, but this relationship was driven by herbivores.

### 4.1 Effect of disturbance on arthropod diversity and abundance

The high arthropod diversity in the most disturbed sites in this study contrasts with previous studies demonstrating a decrease in arthropod diversity with increasing disturbance intensity (Simons et al., 2015; Young et al., 2016). For example, agricultural activities and urbanization can significantly reduce canopy arthropod diversity in temperate forests (Tovar‐Sánchez et al., 2004). Frequent fire is also known to reduce arthropod richness in temperate (Moretti et al., 2006) as well as tropical forests (Little et al., 2013) with fire frequency driving recovery time and ultimately influencing arthropod composition and diversity. Deforestation and logging can also reduce arthropod diversity in temperate (Simard & Fryxell, 2003) and tropical forests (Watt et al., 2002). In contrast, a rich body of literature demonstrates, similarly to our study system, that disturbance can be a potential driver of an increase in arthropod diversity. For example, in temperate regions, trampling, fire, and blowout can increase arthropod and plant diversity (Brunbjerg et al., 2015). In tropical forests in Cote d'Ivoire, high ant species richness was reported in burnt sites over time (Kone et al., 2018). Similar results are also reported elsewhere even though in this system only the recruitment stage experienced disturbance (Floren & Deeleman‐Reinhold, 2005). The positive influence of disturbance on species diversity is well framed by Hutchinson's plankton paradox (Hutchinson, 1961). The random variation in environmental conditions, driven by fires and other chronic anthropogenic disturbances can sustain high diversity by limiting competitive exclusion due to a few species becoming dominant in their preferred environment. Recurrent disturbance can prevent dominant species from establishing long enough to reproduce abundantly, establish themselves, and dominate. However, the same process will directly limit species abundance. Alternatively, such high arthropod diversity in disturbed sites may be explained by the intermediate disturbance hypothesis (Connell, 1978; Fox, 2013). Disturbance in some of our sites may be moderate enough to have limited influence on abundance while creating habitat heterogeneity for niche diversification that supports a higher diversity of arthropods.

### 4.2 Effect of plant diversity on arthropod diversity and abundance

Plant diversity has no significant association with arthropod diversity. However, arthropod abundance increased with plant diversity suggesting biomass‐based bottom‐up influence of plant communities. The lack of bottom‐up significant association between plant diversity and arthropod diversity is in contrast with previous studies (Haddad et al., 2009; Zanuncio et al., 2001) but could be explained by other studies that suggest phylogenetic diversity and not species richness that has a direct effect on arthropod diversity (Staab et al., 2021). The association between plant diversity and arthropod diversity would therefore be an indirect effect mediated by plant phylogenetic diversity, which accounts for the evolutionary relatedness between species. We found a significant effect of plant diversity on the diversity and abundance of predator arthropods. This is consistent with predictions from the enemy hypothesis that high predator diversity will exert a top‐down control on herbivore composition and diversity which in turn will limit its top‐down effect on plants (Staab & Schuldt, 2020). This regulation is facilitated through a top‐down effect: herbivores allowing predators to diversify and increase in number and predators exerting an effect on herbivores to regulate their diversity and numbers (Dassou et al., 2017).

### 4.3 Effect of the arthropod diversity and abundance on the herbivory rate

Herbivory rate was significantly positively associated with arthropod diversity. This interaction is explained by the fact that a high arthropod diversity implies a wide variety of specific herbivore species as well as generalists. Thus, a large proportion of plants in the environment are selected as hosts and sources of food for specialist as well as generalist herbivores. Our results contrast with previous studies that report a negative association between arthropod diversity and herbivory rates (van Klink et al., 2015). The theory of associational resistance (Jactel et al., 2006) predicts a negative influence of plant diversity on herbivory rates due to increased host search time for herbivore in diverse communities. High plant diversity can disrupt the movement of insects, especially specialist herbivores, thereby increasing the likelihood of extinction and decreasing herbivory (Rossetti et al., 2017). In contrast, low plant diversity can easily expose herbivore arthropods (Kelleher & Choi, 2020). However, our study demonstrates heterogeneity in the response of arthropod herbivory to plant diversity, which is driven by habitat quality or harshness.

We observed a significant positive correlation between plant diversity and herbivory rates in the dry Sudanian zone but not in the moist Sudano‐Guinean zone indicating that bottom‐up control in trophic systems is more likely in harsh environmental conditions or poor habitats. Plant diversity can positively influence herbivory rate when they are dominated by generalist herbivores which consume a wide range of plant species. Therefore, a decrease in herbivory rate could also be attributed to a direct loss of generalist herbivore species and/or a reduced abundance of herbivores. In contrast, despite the significant effect of arthropod diversity on herbivory rate, we found no significant association between arthropod abundance and herbivory rate. Because most of the arthropod species sampled in our study are ants which are predators, an increase in arthropod abundance is expected to have minimal effect on plant herbivory. In addition, if the dominant ant community exerts stronger pressure on the limited population of arthropod herbivores, this is expected to further reduce herbivory rates.

## 5 CONCLUSION

Our study showed a decoupled response of arthropod diversity and abundance to disturbance. Disturbance had a significant positive effect on arthropod diversity, but a negative effect on abundance. Our study also revealed that herbivory rate was positively associated with arthropod diversity. High arthropod diversity implies a high diversity of specialist and generalist herbivores and therefore higher herbivory rates. We also demonstrated a significant direct positive influence of plant diversity on herbivory rates which provides counter‐support for the theory of associational resistance but highlights the context dependence of biotic interactions. Finally, we found no significant bottom‐up effect of the plant diversity on arthropod diversity but a significant plant diversity–arthropod abundance relationship. This study illustrates how community‐wide biotic interactions are mediated by chronic anthropogenic disturbance and ecological conditions.

## AUTHOR CONTRIBUTIONS

**Orou G. Gaoue:** Conceptualization (lead); funding acquisition (lead); investigation (equal); methodology (lead); project administration (lead); software (equal); supervision (lead); validation (lead); writing – review and editing (equal). **Hermann Bassoki:** Data curation (lead); formal analysis (equal); investigation (lead); methodology (supporting); software (equal); visualization (equal); writing – original draft (equal). **Anicet G. Dassou:** Conceptualization (supporting); data curation (equal); investigation (equal); methodology (equal); supervision (supporting); writing – original draft (equal); writing – review and editing (equal).

## CONFLICT OF INTEREST STATEMENT

The authors declare no competing or conflicting interests.

## Data Availability

Data and R script used for the statistical analyses are publicly published on FigShare, an open‐access data and code repository (Gaoue et al., 2023) (https://doi.org/10.6084/m9.figshare.24001722.v1).
